# Cm(III) retention by calcium silicate hydrate (C-S-H) gel and secondary alteration phases in carbonate solutions with high ionic strength: A site-selective TRLFS study

**DOI:** 10.1038/s41598-019-50402-x

**Published:** 2019-10-03

**Authors:** Jan-Martin Wolter, Katja Schmeide, Nina Huittinen, Thorsten Stumpf

**Affiliations:** Helmholtz-Zentrum Dresden - Rossendorf, Institute of Resource Ecology, Bautzner Landstraße 400, 01328 Dresden, Germany

**Keywords:** Geochemistry, Geochemistry

## Abstract

We studied the Cm(III) retention by calcium silicate hydrate (C-S-H), portlandite (Ca(OH)_2_) and their alteration products calcite, vaterite, and aragonite in high ionic strength carbonate-containing solutions representing specific formation waters. For this, we synthesized C-S-H gels with calcium to silicon (C/S) ratios of 1.0 and 2.0 in the absence and presence of Cm(III), resulting in Cm(III)-free and Cm(III) doped C-S-H gel, respectively. For phase identification purposes we applied X-ray diffraction (XRD) while for the identification of the Cm(III)/C-S-H binding mode we applied site-selective time-resolved laser-induced luminescence spectroscopy (TRLFS). The stability of Cm(III) doped phases under repository-relevant conditions was evaluated by studying the time-dependent release of Cm(III) from the Cm(III) doped C-S-H gel into leaching solutions containing 0.02 M NaHCO_3_ or 2.5 M NaCl/0.02 M NaHCO_3_ over 60 d. Speciation changes of Cm(III) due to leaching were followed with TRLFS while C-S-H structure alterations and secondary phase formation were monitored with XRD. From the results it could be concluded that Cm(III) is not mobilized by aqueous carbonate but either remains incorporated in the C-S-H structure and portlandite or becomes partially re-immobilized into secondary CaCO_3_ phases. The presence of NaCl led to an accelerated conversion of metastable secondary CaCO_3_ phases into calcite.

## Introduction

Concrete will be used as part of the multi-barrier system in a deep geological nuclear waste repository to ensure mechanical stability and sealing of disposal tunnels against formation water.

Concrete contains hardened cement paste (HCP), a mixture of mineral phases such as calcium silicate hydrate (C-S-H), portlandite, and calcium aluminate hydrates^1^. HCP and especially C-S-H are known to immobilize tri-, tetra- and hexavalent actinides such as Cm(III), Am(III), Np(IV), Pu(IV) and U(VI) potentially released from spent nuclear fuel (SNF)^2–6^. C-S-H is a sheet silicate consisting of polyhedral CaO planes, SiO tetrahedra chains or dimers, and interlayers filled with water or cations such as Na^+^, K^+^ or Ca^2+^, similar to a defected 14 Å tobermorite-like structure. C-S-H properties are determined by the C/S ratio that ranges from 1.7 to 0.6 in synthetic C-S-H gel^7^. Above and below these C/S limits further phases such as portlandite (Ca(OH)_2_) and SiO_2(am)_ are formed^7^. Furthermore, the layer-to-layer distance and length of silicate chains of the C-S-H structure are influenced by the C/S ratio. The concrete barriers in a deep geological repository will be in contact with the host rock and thus, also with its pore water. The access of pore water into the shafts of a deep geological repository could potentially lead to a pore water induced concrete corrosion and finally, to a release of radionuclides from the technical containment. The alteration of concrete leads to release of NaOH, KOH, and Ca(OH)_2_ which shifts the pH of the pore water towards the hyperalkaline region (pH > 12)^8^, which in turn can alter the retention potential of host rock minerals towards radionuclides as shown by Philipp *et al*.^9^.

For clay deposits in North Germany^10,11^ as well as for sedimentary bedrocks in Japan^12^ and Canada^13^, taken into account as potential sites for nuclear waste repositories, high ionic strengths pore waters containing up to 4 M Na^+^, 2.8 M Cl^−^, 0.1 M S${{\rm{O}}}_{4}^{2-}$ and 0.06 M HC${{\rm{O}}}_{3}^{-}$ are reported. In dependence on ionic strength and carbonate concentration of the pore water an increased release of Ca(OH)_2_ and the formation of secondary CaCO_3_ phases can influence the concrete stability, pH of the pore water, and actinide retention by the concrete material^14–16^. In detail, this has been shown in a recent study investigating U(VI) doped C-S-H gel exposed to saline carbonate solutions under alkaline conditions, where a significant release of previously incorporated U(VI) from C-S-H gel was observed in addition to a carbonate-induced alteration of the C-S-H structure^14^. Whether such mobilization will occur also for actinides in other oxidation states should, thus, be investigated in detail, to allow predictions of the long-term safety of nuclear waste repositories.

Neutron capture reactions of isotopes in nuclear fuel lead to the formation of various actinide isotopes of Np, Pu, Am, and Cm^17^. The mass contributed by minor actinides (MAs), Am and Cm, in SNF is rather small^18,19^ but their high contribution of alpha, beta, and gamma radiation over a long time period makes their retention by barriers of a deep geological repository mandatory. Pu and the aforementioned MAs can exist as trivalent cations in repository environments, thus, the present study focuses on understanding the interaction of trivalent actinides with C-S-H phases in saline solutions. As representative for the trivalent actinides we have chosen the luminescent cation Cm(III), which enables laser-induced luminescence spectroscopic investigations of the actinide retention mode in the studied systems.

Previous studies addressing Cm(III) uptake in cementitious environments can be found in the literature^2,20^. Most of the studies on interactions of Cm(III) with cementitious materials or on Cm(III) complexation with relevant ligands were either exclusively focused on the Cm(III) uptake and binding mode or performed at near-neutral to alkaline (but not hyperalkaline) conditions. For instance, nonselective time-resolved laser-induced luminescence spectroscopy (TRLFS) investigations of the Cm(III)/HCP system by Stumpf *et al*.^2^ identified Cm(III) mainly incorporated in the C-S-H structure. Based on luminescence data of Cm(III) sorbed on C-S-H gel obtained by nonselective TRLFS by Tits *et al*.^20^, the authors concluded that Cm(III) is either substituted for Ca^2+^ in the C-S-H interlayer or for Ca^2+^ in the Ca octahedral layers. Fanghänel *et al*.^21^ investigated the complex formation of Cm(CO_3_)_n_^3–2n^ in solutions containing between 0 and 6 M NaCl. The authors observed the formation of aqueous Cm(III) carbonate complexes at pH values ≤ 10.

Thus, the objective of the present work was to study the stability of Cm(III) doped C-S-H gel in solutions containing 0.02 M NaHCO_3_ or 2.5 M NaCl/0.02 M NaHCO_3_, representative for formation water present in some claystone deposits, to evaluate the retention capability of a HCP barrier towards Cm(III) under repository-relevant conditions. For this, C-S-H gel samples with two C/S ratios 2.0 and 1.0, representing a portlandite saturated C-S-H system as well as chemically degraded cement paste, were prepared. For the first time, site-selective TRLFS was applied for Cm(III) doped C-S-H gel and complimented by XRD investigations before and after leaching experiments to study potential alterations of the C-S-H structure, the formation of secondary phases as well as potential changes of the Cm(III) binding mode.

## Results and Discussion

### Characterization of Cm(III)-free and Cm(III) doped C-S-H gel before leaching

The exposure of CaO and fumed silica to the alkaline Cm(III) solution led to a Cm(III) uptake of 98.5 and 97.1% into the solid C-S-H phases having C/S ratios of 1.0 and 2.0. This corresponds to retardation coefficients (*R*_d_) of 2.7 × 10^3^ and 1.2 × 10^3^ L/kg (Table 1).

**Table 1:** Amount of Cm(III) incorporated into C-S-H gel in dependence on C/S ratios.

Sample	C/S ratio	Cm(III) [%]	Cm(III) [ppm]	Cm(III) [mol/kg]	*R*_d_ [L/kg]
1	1.0	98.5	28.5	1.15 × 10^−4^	2.7 × 10^3^
2	2.0	97.1	28.1	1.13 × 10^−4^	1.2 × 10^3^

Some previous studies focused on the sorption behavior of trivalent actinides and lanthanides on C-S-H gel^6,20,22,23^. Häußler *et al*.^6^ determined *R*_d_ values of 4 × 10^5^ L/kg for the sorption of Am(III) on C-S-H. *R*_d_ values determined for Eu(III) sorbed on C-S-H phases by Pointeau *et al*.^22^ and Tits *et al*.^20^ ranged between 2.7 × 10^4^ to (6 ± 3) × 10^5^ L/kg, respectively. Tits *et al*. ascribed the high uncertainty in the *R*_d_ values to an extremely strong sorption of Eu(III) on the C-S-H phase and a possible incomplete phase separation during centrifugation leading to the presence of colloids in the supernatant. Since in the present study a centrifuge with only 3,059 × *g* could be used for Cm(III) samples, it is possible that the observed low *R*_d_ values are also caused by an incomplete phase separation. To support this assumption, Eu(III), which is often applied as a non-radioactive analog for trivalent actinides, was used instead of Cm(III). Thus, Eu(III) doped C-S-H phases with C/S ratios of 1.0 and 2.0 were synthesized under conditions identical to our Cm(III) doped C-S-H phases. These samples were centrifuged at 40,000 × *g* for 1 h. Subsequently, the supernatants were analyzed for Eu(III) resulting in *R*_d_ values of 1.6 × 10^6^ L/kg for both C/S ratios. This shows that the low *R*_d_ values for Cm(III) in the present study were indeed caused by an incomplete phase separation. Since the Cm(III) speciation on solid C-S-H particles present in the supernatant should be identical to that on the solid, it is expected that the incomplete phase separation has no influence on recorded XRD patterns or TRLFS spectra.

**Figure 1:**
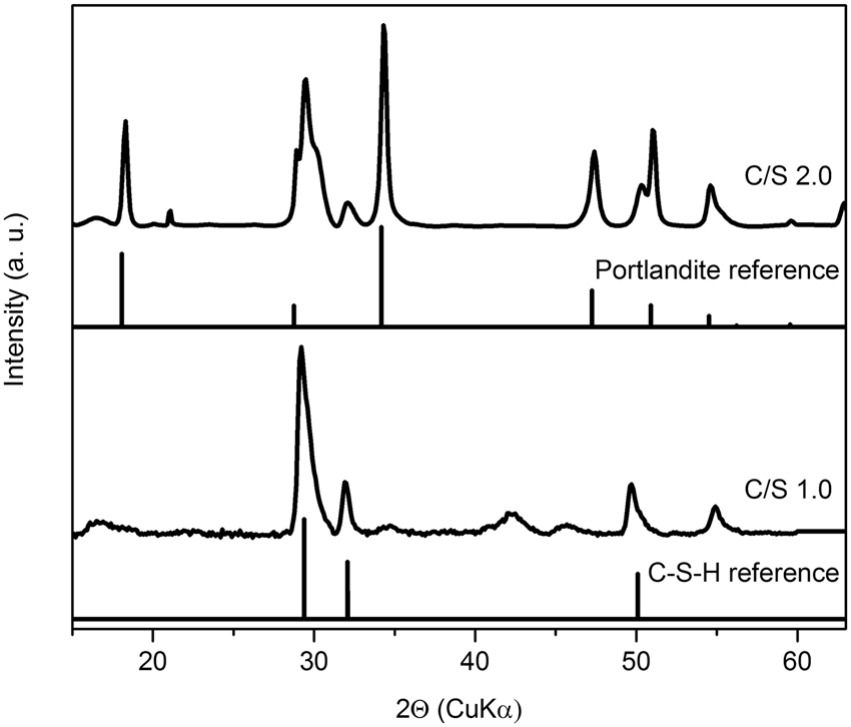
XRD patterns of Cm(III)-free C-S-H samples with C/S ratios of 1.0 and 2.0 in comparison to C-S-H and portlandite references.

Cm(III)-free C-S-H samples were used for phase identification by XRD. These phases show characteristic XRD reflections of the C-S-H structure at 29.1, 32, 42, 46, 50 and 55°2Θ (Fig. 1), identical to those observed for U(VI) doped C-S-H gel^14^. For the C-S-H sample with a C/S ratio of 2.0, in addition, prominent portlandite reflections were detected (Fig. 1). Thermogravimetric analysis (TGA) of a U(VI) doped C-S-H sample with a C/S ratio of 2.0 by Wolter *et al*.^14^, synthesized applying a comparable procedure, showed that at this C/S ratio around 18% of the mass is contributed by portlandite.

**Figure 2:**
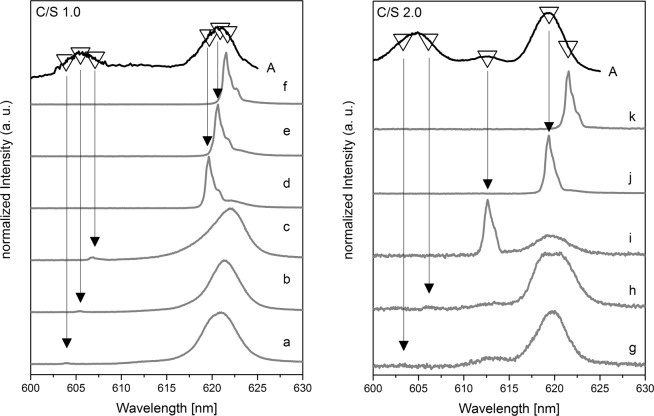
Excitation spectra (A, top black lines) of sample 1 (left) and sample 2 (right) and recorded emission spectra (a-k, gray lines) after excitation at different wavelengths: (**a**) (604.1 nm), (**b**) (605.5 nm), (**c**) (606.9 nm), (**d**) (619.6 nm), (**e**) (620.9 nm), (**f**) (621.5 nm), (**g**) (603.4 nm), (**h**) (606.1 nm), (**i**) (612.6 nm), (**j**) (619.3 nm), (**k**) (621.5 nm).

For the identification of Cm(III) species in the formed solids, site-selective TRLFS was applied. Sample 1 (C/S 1.0) provides a broad excitation spectrum with maxima at 605.5 and 620.9 nm (Fig. 2, left) which are in good agreement with results obtained in nonselective TRLFS studies at 10 K of the Cm(III) incorporation in the C-S-H structure by Tits *et al*.^20^. The authors identified a Cm(III) hot band in C-S-H at 606 nm and a main band around 620 nm that shifted with increasing delay time towards lower energies. A peak deconvolution of the main band was performed and two bands at 618.9 and 620.9 nm with lifetimes of (289 ± 11) µs and (1482 ± 200) µs, respectively, were identified. The twofold split main band was attributed to Cm(III) located in the C-S-H interlayer substituted against Ca^2+^ with 1.4 water molecules in the hydration sphere and Cm(III) incorporated in the polyhedral CaO plane of the C-S-H structure substituted against Ca^2+^ with a total loss of its hydration sphere.

In the present TRLFS study, the excitation of sample 1 (C/S 1.0) between 604.1 and 606.9 nm yields emission lines only in the region around 620.5 nm (Fig. 2, left, a–c). This implies that the broad peak in the excitation spectrum around 605.5 nm (Fig. 2, left) is a hot band of the main band transition (^8^S_7/2_ → ^6^D_7/2_) and not a second non-equivalent Cm(III) species. The obtained emission spectra upon excitation in the hot band region (Fig. 2a–c) are rather broad and asymmetric, pointing towards the presence of more than one species.

After selectively exciting around 620 nm, multiple narrow emission spectra with two visible shoulders at slightly longer wavelengths (red-shifted) become visible (Fig. 2, left, d–f). The magnitude of the red-shift of these two shoulders is 0.5 nm and 1.2 nm from the main peak, independent of the excitation wavelength used. Thus, these shoulders can be ascribed to a partially resolved splitting of the ^8^S_7/2_ ground state.

**Figure 3:**
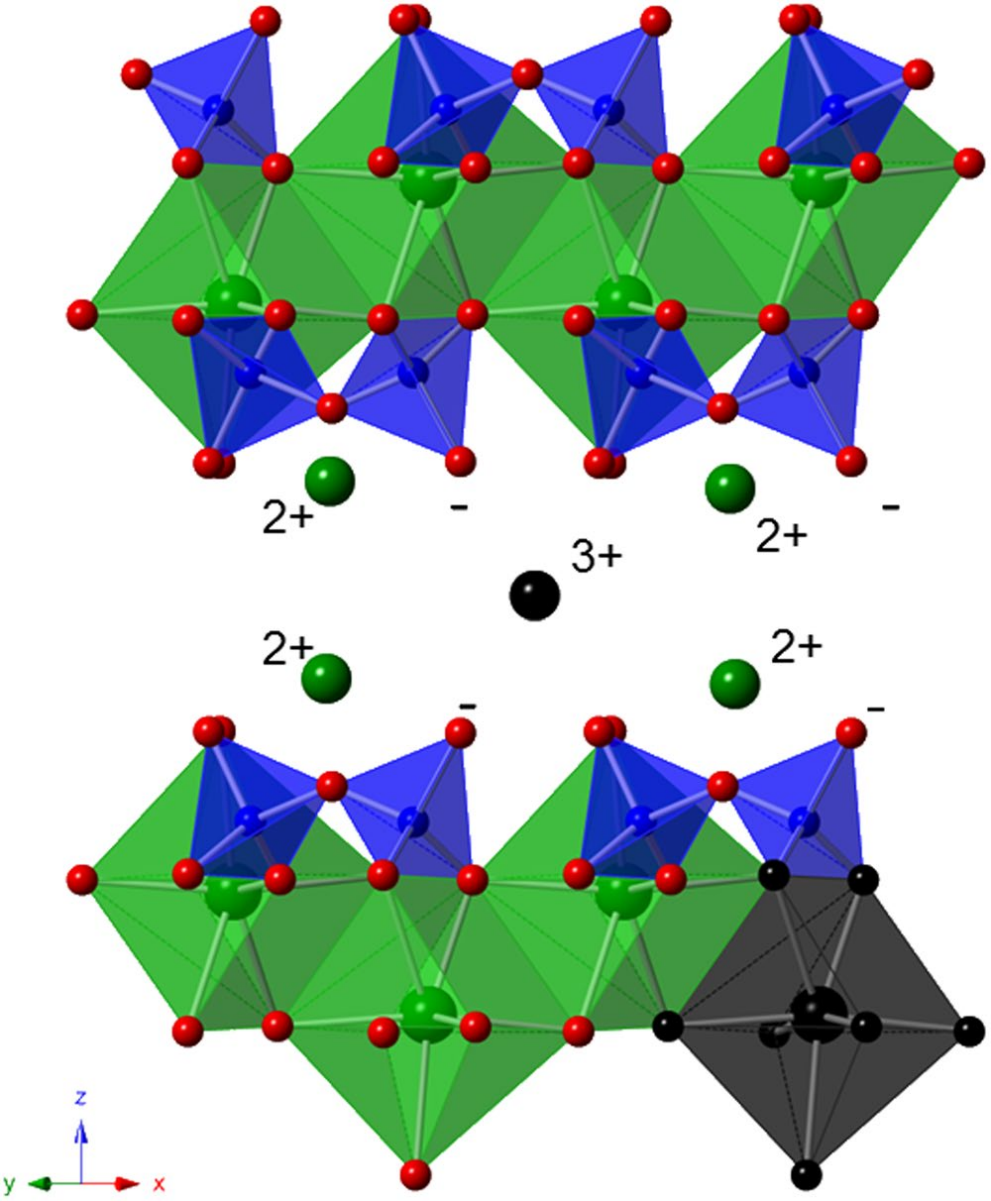
Assumed positions of Cm(III) in the crystal structure of calcium silicate hydrate (calcium (green), silicon (blue), oxygen (red), curium (black)).

The shifting of the emission lines d-f with the applied excitation wavelength is known as luminescence line-narrowing and is visualized in Fig. S1 (supplementary information). For Cm^3+^, line-narrowing effects have been reported for Cm^3+^ incorporation in amorphous grain boundaries in bioapatite^24^ and for Cm^3+^ incorporation in La_1−x_Gd_x_PO_4_ monazite solid solutions^25^. In both studies the line-narrowing was explained by a continuum of related environments arising from the lack of long-range order in the solid structure. Thus, in agreement with these studies, the observed emission line-narrowing in the present study is assigned to variations of the local surrounding of the Cm(III) cation in the semi-crystalline C-S-H structure. A clear distinction between two different species cannot be made, but as already mentioned, indications for the presence of at least two species can be seen in the collected spectroscopic data. This can be confirmed by the averaged luminescence lifetimes of the hot and main band, which follow a bi-exponential decay with averaged lifetimes of (163 ± 111) µs which corresponds to 3.1 water or OH^−^ molecules in the first coordination sphere and (977 ± 51) µs equivalent to a total loss of the hydration sphere (Table S1). The substantial error of 111 μs on the calculated lifetime of one species most probably originates from the low contribution of this species to the composite spectra. These lifetimes imply the presence of a Cm(III) species inside the C-S-H interlayer with 3.1 water or OH^−^ molecules in the first coordination sphere and Cm(III) incorporated into the polyhedral CaO plane with a total loss of the hydration sphere as schematically illustrated in Fig. 3. These results correspond to the findings of Tits *et al*.^20^.

The site-selective luminescence study of sample 2 (C/S 2.0) shows a similar excitation spectrum as recorded for sample 1 with the lower C/S ratio (Fig. 2). However, in addition to the hot band signal around 604.5 nm and the corresponding main band for Cm(III) associated with the C-S-H structure (~619 nm), a weak signal around 613 nm can be discerned in the excitation spectrum, which was not observed in sample 1 (C/S 1.0). Excitation at this peak maximum (612.6 nm) yields an emission signal with a shoulder on the red side (~613.5 nm) (Fig. 2, right, i) and a lifetime of (155 ± 15) µs (Table S1) which corresponds to 3.3 water molecules in the Cm(III) coordination sphere. Please note that these emission lines are strongly red-shifted compared to emission lines of the Cm^3+^ aquo ion (593.8 nm) and the Cm(III) hydroxo complexes (Cm(OH)^2+^ 598.7 nm^26^, Cm(OH)_2_^+^ 603.5 nm^27^, and Cm(OH)_3(aq)_ 607.1 nm^20^) suggesting the formation of a Cm(III)/OH^−^ inner-sphere complex. As previously shown by XRD (Fig. 1) and TGA^14^ investigations, a C-S-H phase with a C/S ratio of 2.0 contains around 18 mass% portlandite in addition to the C-S-H phase. Thus, the additional peak is assumed to arise from Cm(III) association with portlandite. TRLFS investigations of Cm(III) sorbed on HCP/portlandite by Stumpf *et al*.^2^ showed the formation of an inner-sphere Cm(III)/portlandite complex with an emission spectrum at 613.6 nm and a lifetime of (66 ± 1) µs which corresponds to 9 OH^−^ molecules in the first coordination sphere. The peak position corresponds well with that observed in the present study even though the lifetimes are different. This may be due to the different experimental conditions employed in the two studies (here site-selective excitation of Cm(III) in a mixture of dried C-S-H and portlandite at 10 K in comparison to Cm(III) exposed to a portlandite suspension for 30 d excited with 395 nm at room temperature by Stumpf *et al*.^2^). Thus, it is assumed that the amount of H_2_O and OH^−^ in portlandite was higher in the study of Stumpf *et al*., which explains the longer lifetimes observed in the present study. Nonetheless, in agreement with the study by Stumpf *et al*.^2^ we assign the signal at ~613 nm to a Cm(III) inner-sphere complex incorporated in the formed portlandite phase.

As already indicated for sample 1 (C/S 1.0), the emission spectra of sample 2 obtained after selective excitation in the hot band region, especially at λ_ex_ = 606.1 nm (Fig. 2, right, h), shows a very asymmetric emission peak with two clear maxima, corroborating the presence of two non-equivalent Cm(III) species in the C-S-H structure. Furthermore, the lifetime decay analyses after excitation of the C-S-H hot- and main bands of this sample show a bi-exponential decay behavior with averaged lifetimes of (214 ± 28) µs and (928 ± 109) µs (Table S1), corresponding to Cm(III) species with 2 and 0 water molecules, respectively, in the first coordination sphere of the actinide cation.

### Leaching of Cm(III) doped C-S-H gel with a C/S ratio of 1.0

The exposure of sample 1 (C/S 1.0) to solutions that contain either 0.02 M NaHCO_3_ or 2.5 M NaCl/0.02 M NaHCO_3_ over a time period of 14, 30 or 60 d led to a negligible Cm(III) mobilization between 0.01 and 0.5% of the previously incorporated Cm(III) (Table 2).

**Table 2:** Amount of Cm(III) leached from sample 1 (C/S 1.0) and final pH values of 0.02 M NaHCO_3_ or 2.5 M NaCl/0.02 M NaHCO_3_ leaching solutions.

Conditions	Cm(III) leached [M]	Cm(III) leached [%]	pH
NaHCO_3_, 14 d	3.8 × 10^−10^	0.014	10.9 ± 0.2
NaHCO_3_, 60 d	2.4 × 10^−9^	0.086	10.9 ± 0.1
NaCl/NaHCO_3_, 14 d	2.2 × 10^−10^	0.008	10.6 ± 0.3
NaCl/NaHCO_3_, 60 d	1.4 × 10^−8^	0.498	10.7 ± 0.3

Comparable leaching experiments performed with U(VI) doped C-S-H gel samples by Wolter *et al*.^14^ showed a much higher U(VI) release (up to 40%) which was attributed to the formation of Ca_2_UO_2_(CO_3_)_3_ in solution at pH values ≤ 10.4. Cm(III) carbonate complexation studies performed in solutions containing 0 to 6 M NaCl by Fanghänel *et al*.^21^ indicated the presence of Cm(CO_3_)_n_^3–2n^ complexes at pH < 10 and carbonate concentrations ≥10^−4^ M. A comparison of the stability constants at infinite dilution (log *β*^0^) reveals lower stability constants for Cm(CO_3_)_n_^3–2n^ (log *β*^0^_101_ = 8.1 ± 0.3, log *β*^0^_102_ = 13.0 ± 0.6, log *β*^0^_103_ = 15.2 ± 0.4, and log *β*^0^_104_ = 13.0 ± 0.5^21^) compared to Ca_2_UO_2_(CO_3_)_3_(aq) (log *β*^0^_213_ = 30.45 ± 0.35^28^). Therefore, comparable NaHCO_3_ concentrations should lead to a stronger complexation of U(VI) by carbonate compared to Cm(III). Furthermore, differences in the S/L ratio and a higher pH after leaching contribute to the lower Cm(III) release compared to U(VI).

**Figure 4:**
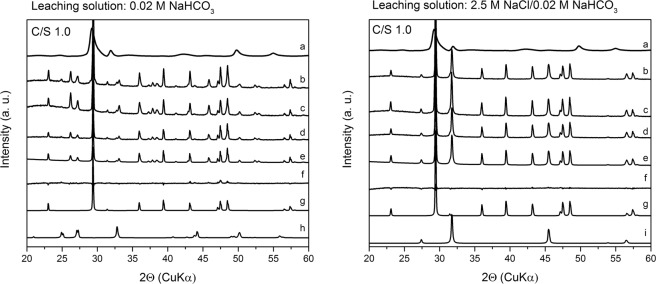
XRD patterns of the Cm(III)-free C-S-H gel with a C/S ratio of 1.0 before and after leaching in 0.02 M NaHCO_3_ (left) and 2.5 M NaCl/0.02 M NaHCO_3_ (right) depending on leaching time: (**a**) (before leaching), (**b**) (after 1 d), (**c**) (after 8 d), (**d**) (after 30 d), (**e**) (calculated after 30 d), (**f**) (difference between measured and calculated spectra after 30 d), (**g**) (calcite), (**h**) (aragonite), (**i**) (halite).

To identify factors such as secondary phase formation that might be responsible for the low Cm(III) release in the present study, XRD analyses and subsequent Rietveld refinements of the XRD patterns of a Cm(III)-free C-S-H gel (C/S 1.0) after leaching in 0.02 M NaHCO_3_ or 2.5 M NaCl/0.02 M NaHCO_3_ were performed (Fig. 4, Table S2).

The results revealed that a part of the C-S-H gel with a low C/S ratio (1.0) is converted into secondary CaCO_3_ phases such as calcite and aragonite in dependence on the composition of the leaching solution. After leaching, C-S-H reflections in the XRD patterns are not detectable anymore, probably due to the higher crystallinity of the CaCO_3_ polymorphs compared to the semi-crystalline C-S-H structure and the partial conversion of C-S-H into calcite and aragonite both resulting in low intensity of C-S-H reflections. The formation of CaCO_3_ polymorphs in C-S-H gels and clinker phases was reported in literature before whereby the proportions of the various CaCO_3_ modifications seem to depend on the sample preparation and carbonation method^14–16,29^.

At a S/L ratio of 10 g/L, a 0.02 M NaHCO_3_ solution could convert a maximum of 23 mol% (cf. Table S3) of sample 1 into CaCO_3_. These 23 mol% Ca of the C-S-H gel with a C/S ratio of 1.0 were converted in average to (67 ± 9)% calcite and (33 ± 9)% aragonite (Fig. 4, Table S2). No clear trend in terms of CaCO_3_ phase conversion was found over 1 month, probably caused by the combination of two effects. On one hand, aragonite should be converted constantly into calcite due to its metastable nature. On the other hand, the rotation of the samples during leaching grinds the samples, creating new C-S-H surfaces that are exposed to the carbonate solution which promotes the formation of metastable aragonite.

If the same sample was leached in a carbonate-containing solution that additionally contained 2.5 M NaCl only calcite was detected (Table S2). Takita *et al*.^30^ reported a similar effect and tentatively attributed this to an increased solubility and subsequent faster recrystallization of the metastable CaCO_3_ polymorphs. Additionally, halite reflections were detected originating from drying the leached C-S-H gel which leads to a crystallization of NaCl residues in the samples.

Despite the clear transformation of a part of the C-S-H structure into secondary phases, the very low Cm(III) release from C-S-H gel points towards either incongruent leaching of the sample, where Cm(III) remains in the C-S-H structure, or a Cm(III)-re-immobilization into the formed secondary CaCO_3_ phases. To trace the association of Cm(III) in the solid mixtures, site-selective TRLFS analyses of the solids after leaching were performed.

**Figure 5:**
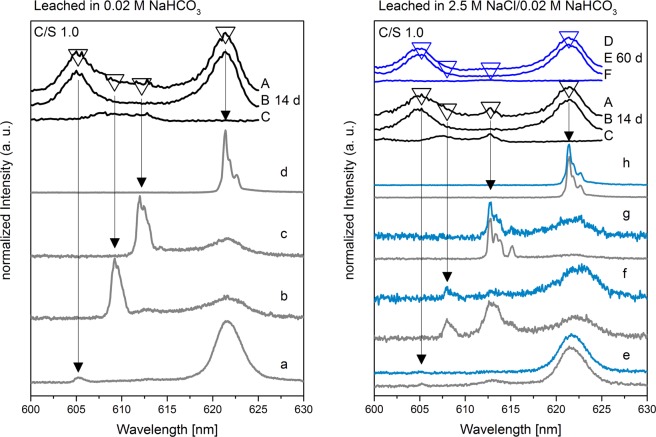
TRLFS spectra of sample 1 (C/S 1.0) after leaching in 0.02 M NaHCO_3_ (left) for 14 d and in 2.5 M NaCl/0.02 M NaHCO_3_ (right) for 14 d (gray lines) and 60 d (blue lines). (**A–C**) Excitation spectra after 14 d (black lines). (**D–F**) Excitation spectra after 60 d (blue lines). (**A,D**) Integrated over the complete emission spectrum. (**B,E**) Integrated over the C-S-H main band (~615–625 nm). (**C,F**) Integrated between the C-S-H hot and main band (~605–615 nm). Recorded emission spectra after excitation at different wavelengths: a (605.2 nm), b (609.2 nm), c (612.2 nm), d (621.4 nm), e (605.2 nm), f (608.0 nm), g (612.8 nm), h (621.4 nm).

The excitation spectrum of sample 1 (C/S 1.0) leached in 0.02 M NaHCO_3_ for 14 d shows a new broad peak between 607.7 and 612.8 nm (Fig. 5, left, A and C) in addition to the Cm(III)/C-S-H hot and main bands at 606 and 620 nm, respectively (Fig. 5, left, A and B). After selective excitation in the region where the new Cm(III) species is detected, threefold split emission spectra (Fig. 5, left, b,c) with lifetimes of (734 ± 55) µs are obtained which corresponds to a total loss of the hydration sphere (Table S4). In order to assign the new Cm(III) species, the obtained data in terms of emission peak position and lifetime are compared to published data for Cm(III) association with the CaCO_3_ polymorphs calcite and aragonite. Marques Fernandes *et al*.^31^ investigated the association of Cm(III) with calcite at a pH value of 12.5. At these solution conditions, the authors found a Cm(OH)^2+^ species incorporated within the calcite structure with an emission band at 608.2 nm and a lifetime of (477 ± 25) µs, Table 3. Other calcite species were reported with emission peak maxima at 606.2 nm and 620.3 nm^31^. These peak positions overlap with the hot band and main band transitions of the Cm(III)/C-S-H species in the present study and thus, we cannot make any assignment to Cm(III)/calcite species in these emission wavelength ranges. Cm(III) incorporated in aragonite was found by Schmidt *et al*.^32^ to result in a main band transition at 612.7 nm, a corresponding hot band transition at 607.5 nm and a lifetime of (637 ± 77) µs (Table 3). Even though the signal obtained in the present study for the presumed Cm(III)/CaCO_3_ species is very broad, the two local maxima at 607.7 nm and 612.8 nm can be distinguished in the excitation spectrum integrated between 605 and 615 nm (Fig. 5, left, C; Fig. S2). Thus, it is likely that Cm(III) indeed is incorporated within the formed aragonite phase as a result of the C-S-H phase conversion. However, the broad nature of the excitation peak recorded in the present study implies that other sorption/incorporation processes may contribute to the Cm(III) speciation after leaching in 0.02 M NaHCO_3_ solutions which we cannot conclusively assign based on the recorded luminescence data.

**Table 3:** Band positions and lifetimes of Cm(III) species in relevant reference systems.

Cm(III) species	Band position [nm]	Lifetimes [µs]
Cm(III) surface sorbed on calcite, pH 8–12.5^31^	606.2	386 ± 40 (298 K)
CmOH^2+^ incorporated in calcite, pH 12.5^31^	608.2	477 ± 25 (298 K)
Cm(III) incorporated in calcite, pH 8–12.5^31^	620.3	1874 ± 200 (298 K)
Cm(III) incorporated in vaterite^33^	612.1	1802 ± 216 (<20 K)
	619.1	2569 ± 308 (<20 K)
Cm(III) incorporated in aragonite^32^	607.5	637 ± 77 (<20 K)
	612.7	637 ± 77 (<20 K)
Cm(III) incorporated in portlandite (Ca(OH)_2_)^2^	613.6	62 ± 8 (298 K)
Cm(III) in C-S-H interlayer^20^	618.9	289 ± 11 (10 K)
Cm(III) in C-S-H polyhedral CaO plane^20^	620.9	1482 ± 200 (10 K)

Excitation spectra of sample 1 (C/S 1.0) leached for 14 and 60 d in 2.5 M NaCl/0.02 M NaHCO_3_ are presented in Fig. 5, right, as black lines A-C and blue lines D-F, respectively. Although XRD confirmed the exclusive presence of calcite, TRLFS shows multiple low-intensity emission lines between 608.0 and 612.8 nm (Fig. 5, right, f,g, gray lines). After excitation at 612.8 nm, a splitting of the emission line in four separated peaks is observed (Fig. 5, right, g, gray line). This fourfold splitting was also observed by Schmidt *et al*.^32^ after excitation of Cm(III)-containing aragonite at 612.7 nm caused by a fourfold ground state splitting of the ^8^S_7/2_ state. The decreased intensity of these emission lines after 60 d of leaching in 2.5 M NaCl/0.02 M NaHCO_3_ (Fig. 5, right, f,g, blue lines) at 608.0 and 612.8 nm indicates that these emission lines are caused by Cm(III) incorporation into aragonite that is probably present in amounts below the XRD detection limit. The aragonite phase is converted into calcite over time due to its metastable nature, thus, causing the decrease of the Cm(III)/aragonite emission lines. However as discussed above, due to the overlap of the signals of Cm(III) incorporated in calcite with those of Cm(III)/C-S-H gel, we cannot detect this species in the current system.

### Leaching of Cm(III) doped C-S-H gel with a C/S ratio of 2.0

To investigate the influence of the portlandite phase on the stability of the Cm(III)/C-S-H system, sample 2 (C/S 2.0) was leached in either 0.02 M NaHCO_3_ or 2.5 M NaCl/0.02 M NaHCO_3_. Comparable to sample 1, the leaching of sample 2 leads only to a very low Cm(III) release between 0.05 and 0.7% of the previously incorporated Cm(III) (Table 4).

**Table 4:** Amount of Cm(III) leached from sample 2 (C/S 2.0) and final pH values of 0.02 M NaHCO_3_ or 2.5 M NaCl/0.02 M NaHCO_3_ leaching solutions.

Conditions	Cm(III) leached [M]	Cm(III) leached [%]	pH
NaHCO_3_, 14 d	1.2 × 10^−9^	0.045	12.1 ± 0.01
NaHCO_3_, 60 d	2.0 × 10^−9^	0.075	12.2 ± 0.01
NaCl/NaHCO_3_, 14 d	4.6 × 10^−9^	0.170	12.0 ± 0.01
NaCl/NaHCO_3_, 60 d	2.0 × 10^−8^	0.737	12.1 ± 0.01

**Figure 6:**
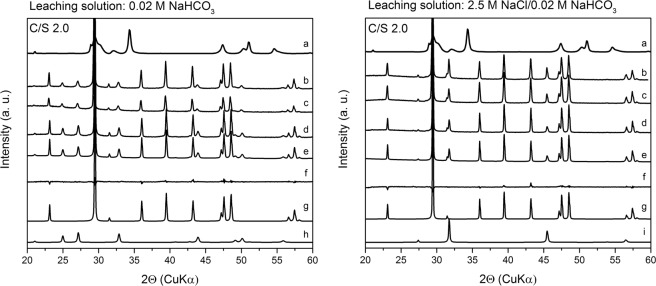
XRD patterns of the Cm(III)-free C-S-H gel with a C/S ratio of 2.0 before and after leaching in 0.02 M NaHCO_3_ (left) and 2.5 M NaCl/0.02 M NaHCO_3_ (right) depending on leaching time: (**a**) (before leaching), (**b**) (after 1 d), (**c**) (after 8 d), (**d**) (after 30 d), (**e**) (calculated after 30 d), (**f**) (difference between measured and calculated spectra after 30 d), (**g**) (calcite), (**h**) (vaterite), (**i**) (halite).

To get a deeper understanding of the phase conversion of portlandite and C-S-H in these leaching solutions, XRD patterns of a Cm(III)-free C-S-H gel with a C/S ratio of 2.0 leached in 0.02 M NaHCO_3_ and 2.5 M NaCl/0.02 M NaHCO_3_ were recorded (Fig. 6).

Under the assumption that the 0.02 M NaHCO_3_ solution would react completely with sample 2 (C/S 2.0) at a S/L ratio of 10 g/L, a maximum of 18% of sample 2 might be converted into CaCO_3_ phases. The results of the Rietveld analyses of the XRD patterns suggest that these 18% of sample 2 are converted into an average of (84 ± 3)% calcite and (16 ± 3)% vaterite after leaching in 0.02 M NaHCO_3_ while reflections of aragonite are missing (Fig. 6, Table S5). The additional presence of 2.5 M NaCl leads to the exclusive formation of calcite, similar to the results previously observed for sample 1 (C/S 1.0) (cf. Tables S2 and S5).

**Figure 7:**
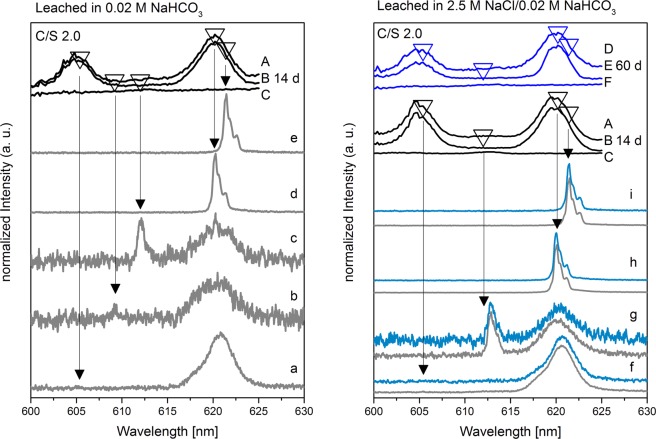
TRLFS spectra of sample 2 (C/S 2.0) after leaching in 0.02 M NaHCO_3_ (left) for 14 d and in 2.5 M NaCl/0.02 M NaHCO_3_ (right) for 14 d (gray lines) and 60 d (blue lines). (**A–C**) Excitation spectra after 14 d (black lines). (**D–F**) Excitation spectra after 60 d (blue lines). (**A,D**) Integrated over the complete emission spectrum. (**B,E**) Integrated over the C-S-H main band (~615–625 nm). (**C,F**) Integrated between the C-S-H hot and main band (~605–615 nm). Recorded emission spectra after excitation at different wavelengths: a (605.4 nm), b (609.2 nm), c (612.0 nm), d (620.2 nm), e (621.4 nm), f (605.4 nm), g (612.8 nm), h (620.0 nm), i (621.4 nm).

In contrast to the leached sample 1 (C/S 1.0), sample 2 (C/S 2.0) leached in 0.02 M NaHCO_3_ shows no emission line between 608 and 609 nm (Fig. 7, left, b), but a weak emission line around 612.0 nm (Fig. 7, left, c). This emission line differs from the emission lines of Cm(III) incorporated into aragonite, observed for sample 1 (C/S 1.0) after leaching in 0.02 M NaHCO_3_ (cf. Fig. 5b).

The new emission line at 612.0 nm is either caused by Cm(III) that is still incorporated into portlandite (612.6 and 613.5 nm, τ: 155 µs, Table S1) or Cm(III) incorporated into the secondary phase vaterite (612.1 nm, τ: 1802 µs^33^).

Since the intensity of the emission line at 612.0 nm (Fig. 7, left, c) is near the noise level after 5000 accumulations, a lifetime analysis with the applied 100 accumulations was not feasible. Since Cm(III) incorporated into vaterite would possess a very long lifetime of 1802 µs as observed by Schmidt *et al*.^33^, it is concluded that the band at 612.0 nm (Fig. 7, left, c) is caused by Cm(III) remaining in portlandite.

The excitation and emission spectra of sample 2 (C/S 2.0), recorded after 14 and 60 d of leaching in 2.5 M NaCl/0.02 M NaHCO_3_, are shown in Fig. 7 (right). The presence of the emission line around 612.8 nm (Fig. 7, right, g, gray line) that also possesses a low intensity and a rather short lifetime (τ: (312 ± 39) µs, Table S6) indicates that this band is caused by Cm(III) incorporated in portlandite instead of metastable vaterite. Furthermore, the persistence of this emission line after 60 d of leaching (Fig. 7, right, g, blue line) underlines this assumption.

Combined TRLFS and XRD results suggest that the majority of Cm(III) remains in the C-S-H and portlandite phases after leaching in saline carbonate-containing solutions. A minor incorporation or sorption of Cm(III) in calcite cannot be excluded due to the main band overlap of the Cm(III)/calcite and Cm(III)/C-S-H system.

## Conclusions

For the first time, site-selective TRLFS investigations (at about 10 K) on Cm(III)-containing C-S-H gel were successfully applied. The additional combination with batch leaching experiments and XRD studies resulted in important information about the Cm(III)/C-S-H binding mode, C-S-H structure alteration, and Cm(III) retention by cementitious materials in complex high ionic strength pore waters.

In the synthesized C-S-H gel at least two Cm(III) species were identified: (i) Cm(III) surrounded by two to three water molecules in the first coordination sphere substituted against Ca^2+^ from the C-S-H interlayer and (ii) Cm(III) incorporated in the polyhedral CaO plane of the C-S-H lattice with a complete loss of the hydration sphere. This is in agreement with the study of Tits *et al*.^20^. In addition, a luminescence line-narrowing effect was detected during the site-selective TRLFS measurements of the present study which indicates variations of the local Cm(III) coordination environment in C-S-H gel. That means, between the two binding sites for Cm(III), there are most probably numerous, chemically similar binding sites for Cm(III) which can be attributed to the amorphous to semi-crystalline structure of the C-S-H gel. In addition, C-S-H gel with a C/S ratio of 2.0 showed a co-incorporation of Cm(III) into portlandite.

The Cm(III) mobilization from Cm(III) doped C-S-H gel in the leaching solutions 0.02 M NaHCO_3_ or 2.5 M NaCl/0.02 M NaHCO_3_ was very low (0.1 to 0.7% after 60 d) regardless of C/S ratio, ionic strength, carbonate presence, or leaching time.

XRD investigations of the C-S-H samples leached in 0.02 M NaHCO_3_ revealed that C-S-H gel and portlandite are partly converted into secondary CaCO_3_ phases such as calcite and aragonite (C/S 1.0) or calcite and vaterite (C/S 2.0). After leaching in 2.5 M NaCl/0.02 M NaHCO_3_ exclusively calcite was detected as secondary phase.

Site-selective TRLFS investigations after leaching showed that Cm(III) was still incorporated in C-S-H gel and in addition, either partially incorporated into the secondary phase aragonite (C/S 1.0) or still incorporated by portlandite (C/S 2.0). An incorporation of Cm(III) into calcite, which was clearly identified in the leached C-S-H gel by XRD, is expected but could not be verified by TRLFS due to the overlap of the luminescence signals of Cm(III) incorporated in calcite with those of Cm(III) incorporated in C-S-H gel. An incorporation of Cm(III) into calcite is also expected after the conversion of the metastable CaCO_3_ phases aragonite and vaterite into the stable phase calcite.

For the conditions applied in the present study (S/L ratio of 10 g/L, pH ≥ 10.6) and thus, also for conditions foreseen for nuclear waste repositories containing protective barriers of concrete (very high S/L ratios), the results of leaching experiments and site-selective TRLFS showed that an almost complete retention of Cm(III) by cementitious materials and their alteration phases can be expected even in the presence of carbonate-containing solutions with increased ionic strengths.

## Materials and Methods

### Materials

C-S-H gel syntheses and leaching experiments were performed in an inert gas glove box (N_2_ atmosphere, CO_2_ < 3.8 ppb) at 25 °C using degassed deionized water (18 MΩ cm; mod. Milli-RO/Milli-Q-System, Millipore, Schwalbach, Germany).

A 4 × 10^−6^ M ^248^Cm stock solution in 0.1 M HClO_4_ was used for the synthesis of Cm doped C-S-H gel. *Caution!* ^248^*Cm is a radionuclide with a half-life of 3.48* × 10^5^ *years, decaying through α-emission (92%) and spontaneous fission (8%). The use of* ^248^*Cm requires the appropriate infrastructure and personnel trained in the handling of alpha-emitting isotopes*.

For C-S-H synthesis, NaOH (p.a., Roth, Karlsruhe, Germany), fumed silica (AEROSIL 300, Evonik, Essen, Germany) and carbonate-free CaO (anhydrous, trace metals basis, Sigma-Aldrich, St. Louis, Missouri, USA) were used. To ensure a carbonate-free NaOH solution, surface carbonate was removed by washing the NaOH pellets several times with degassed deionized water in a Büchner funnel under inert gas atmosphere. The total inorganic carbonate concentration of the prepared NaOH solution, determined with a multi-N/C 2100 S (Analytik Jena, Jena, Germany), was below 100 µM.

### Synthesis of C-S-H gel in absence and presence of Cm(III)

For the synthesis of Cm(III) doped C-S-H gel, 3.6 mL of a 4 × 10^−6^ M Cm(III) stock solution and 1.4 mL deionized water were added to 120 mg of a mixture of carbonate-free CaO and fumed silica, resulting in an initial Cm(III) concentration of 2.88 × 10^−6^ M and a S/L ratio of 24 g/L. The ratios of CaO and fumed silica were varied according to the targeted C/S ratios 1.0 and 2.0. Immediately, the suspensions were homogenized and carbonate-free NaOH was added to achieve a NaOH concentration of 0.294 M. After 14 d of shaking the samples end-over-end with a rotator SB2 (Stuart, Staffordshire, UK), the phases of the suspensions were separated via centrifugation at 3,059 × *g* for 1 h (EBA 280, Hettich Lab Technology, Tuttlingen, Germany). The supernatant solutions were analyzed for Cm(III) by liquid scintillation counting (LSC) using a TriCarb 3100TR counter (Perkin Elmer, Freiburg, Germany) and Ultima Gold™ (Perkin Elmer) as scintillation cocktail. Retardation coefficients (*R*_d_) were determined according to Eq. (1)

1 $${R}_{{\rm{d}}}=\frac{{{\rm{c}}}_{0}-{{\rm{c}}}_{{\rm{eq}}}}{{{\rm{c}}}_{{\rm{eq}}}}\cdot \frac{{\rm{V}}}{{\rm{m}}\,}[{\rm{L}}/{\rm{kg}}]$$

where c_0_ and c_eq_ [mol/L] are the initial and equilibrium Cm(III) concentration, V [L] represents the solution volume and m [kg] the C-S-H mass.

The synthesized Cm(III) doped C-S-H gel samples were stored as wet pastes. Cm(III)-free C-S-H gel samples used in the XRD investigations were synthesized accordingly, but 3.6 mL deionized water were added instead of the Cm(III) stock solution.

### Batch leaching experiments

Wet pastes of Cm(III) doped and Cm(III)-free C-S-H samples were equilibrated in 0.02 M NaHCO_3_ or in 2.5 M NaCl/0.02 M NaHCO_3_ at a S/L ratio of 10 g/L by shaking the samples end-over-end with a rotator SB2. After 14 to 60 d, the phases of the suspensions were separated by centrifugation (3,059 × *g*). Each supernatant solution was analyzed for Cm(III) concentration and final pH values were determined. The pH values after leaching were measured with a pH meter (inoLab pH 720, WTW, Weilheim, Germany) with a SenTix^®^Mic pH microelectrode (WTW), calibrated using standard buffer solutions (pH: 6.865, 9.180 and 12.454) (WTW) and corrected for high ionic strengths (due to 2.5 M NaCl) using a method reported by Altmaier *et al*.^34^. Leached C-S-H gel samples were stored as wet pastes for further characterization. All leaching experiments were performed as duplicates.

### Analytical techniques

#### Time-resolved laser-induced luminescence spectroscopy (TRLFS)

For the evaluation of the Cm(III) binding mode on C-S-H and formed secondary phases, site-selective TRLFS was applied. The method allows for selective excitation of non-equivalent Cm(III) species in the investigated system by tuning the excitation wavelength (λ_ex_) to match the transition energy between the ^8^S_7/2_ ground state and the ^6^D_7/2_ emitting state of the individual species. The excited ^6^D_7/2_ state of the curium ion undergoes crystal field splitting of about 300–600 cm^−1^ due to ligand interactions, giving rise to a maximum of four crystal field states (hot bands). This crystal field splitting is rather small resulting in thermally populated crystal field states at ambient temperatures. In systems with multiple non-equivalent curium species, some overlap of hot band transitions of energetically lower-lying species with the main transition of higher-lying species, may take place. In systems that possess a semi-crystalline character, such as the C-S-H structure investigated in the present study, the environment of an incorporated luminescent species is rather ill-defined, resulting in a multitude of chemically similar, but not identical Cm(III) environments in the amorphous bulk. These non-equivalent Cm(III) species will have slightly different energy states resulting in a multitude of overlapping emission lines and further in so called luminescence line-broadening of the collected excitation spectra.

The excitation spectra of the Cm(III) doped C-S-H gel samples were recorded by varying the excitation wavelength in 0.1 or 0.2 nm steps between 600 and 630 nm. The emission signal obtained at each chosen wavelength was integrated and plotted against the applied wavelengths. High-resolution emission spectra were thereafter collected at the excitation peak maxima, corresponding to individual Cm-species (or hot bands), associated with the C-S-H structure or their alteration products. Finally, lifetime analyses of the Cm(III) species provide information about the number of water or hydroxide molecules in the first Cm(III) coordination sphere, which can be calculated according to an equation developed by Horrocks and Sudnick^35^ for Eu(III) and later applied to Cm(III) by Kimura and Choppin (2)^36^

2 $$n({{\rm{H}}}_{2}{\rm{O}}/{{\rm{OH}}}^{-})=0.65{k}_{{\rm{obs}}}-0.88$$

where *k*_obs_ is the decay rate (reciprocal lifetime) [ms^−1^] of the excited state and *n*(H_2_O/OH^−^) represents the number of coordinated water molecules or hydroxyl ions.

In this work, for TRLFS measurements the dried solid samples were transferred into an Al sample holder and covered with a quartz plate (Qiopitq, Göttingen, Germany). The site-selective TRLFS measurements were performed with a pulsed Nd:YAG (Continuum Surelite II, USA) pumped dye laser set-up (Radiant Dyes Narrow Scan K). A combination of laser dyes such as sulforhodamine B, rhodamine B, and rhodamine 101 were used to enable tuning of the excitation wavelength between 600 and 630 nm. To improve the resolution of the spectra, the samples were cooled with a helium-refrigerated cryostat below a temperature of 12 K. The emission signal was collected by a fiber coupled optical multi-channel system consisting of a polychromator with 600 and 1200 lines/mm gratings and an intensified CCD camera model iStar (Andor, Belfast, Ireland). Emission measurements were accumulated over 5,000 spectra while excitation and time-resolved measurements were accumulated over 100 spectra. Luminescence lifetimes were monitored as a function of delay time between 10 and 1,570 µs in 40 µs steps. The excitation spectra were energy corrected for each wavelength using the average energy collected with an optical power meter (Newport 1918-R).

#### Powder X-ray diffraction (XRD)

XRD analyses of Cm(III)-free C-S-H gel samples before and after leaching were performed to characterize the solid phases including secondary phases formed due to leaching. The diffractograms were collected with a MiniFlex 600 diffractometer (Rigaku, Tokyo, Japan) equipped with a Cu *Kα* X-ray source (40 keV/15 mA operation for X-ray generation) and the D/teX Ultra 1D silicon strip detector in the Bragg-Brentano *θ*-2*θ* geometry at a scanning speed of 0.6° per min. The samples were mounted as wet pastes on a zero-background Si sample holder and stored for several minutes in an inert gas atmosphere to remove excess water from the samples and thus, to minimize carbonation of the samples during measurements. The subsequent Rietveld analysis of diffractograms was done with the program PDXL 2 (Rigaku) and the ICDD PDF-4+ 2016 database (C-S-H phase (database card number 00-033-0306), portlandite (database card number 01-083-4600), calcite (database card number 01-083-4601), aragonite (database card number 01-075-9985), vaterite (database card number 04-017-8634) and halite (database card number 00-005-0628)).

## Supplementary information

Supporting Information

## Data Availability

The datasets generated during and/or analyzed during the current study are available from the corresponding author on reasonable request.
