# Supplementary material for: Cm(III) retention by calcium silicate hydrate (C-S-H) gel and secondary alteration phases in carbonate solutions with high ionic strength: A site-selective TRLFS study
Source: Sci Rep. 2019 Oct 3;9:14255. doi: 10.1038/s41598-019-50402-x (PMC6776662; doi:10.1038/s41598-019-50402-x)
Supplement: Supplementary file 1 — Supporting Information [file 41598_2019_50402_MOESM1_ESM.pdf]

Cm(III) retention by calcium silicate hydrate (C-S-H) gel and secondary alteration phases in carbonate solutions with high ionic strength: A site-selective TRLFS study

Jan-Martin Wolter, Katja Schmeide*, Nina Huittinen, Thorsten Stumpf

Helmholtz-Zentrum Dresden - Rossendorf, Institute of Resource Ecology, Bautzner Landstr. 400, 01328 Dresden, Germany

* Corresponding author. Tel.: +49 351 260 2436; fax: +49 351 260 3553.
E-mail address: k.schmeide@hzdr.de (Katja Schmeide).

Supporting Information

Table S1
Cm(III) emission band positions and lifetimes of sample 1 (C/S 1.0) and sample 2 (C/S 2.0) at 10 K before leaching.

Sample	λ_{ex} [nm]	Band positions [nm]	Lifetime ± 2σ [μs]	Species
1	604.1	619.8, 621.2 (d)		C-S-H hot band
	605.5	621.2	163 ± 132	C-S-H hot band
			964 ± 48	
	606.9	622.1		C-S-H hot band
	619.6	619.6, 620.1, 620.8 (t)		C-S-H main band
	620.9	620.6, 621.2, 621.9 (t)	163 ± 90	C-S-H main band
			989 ± 54	
	621.5	621.5, 622.1, 622.8 (t)		C-S-H main band
2	603.4	619.9		C-S-H hot band
	606.1	618.5, 621.0 (d)	227 ± 40	C-S-H hot band
			1068 ± 175	
	612.6	612.6, 613.5 (d)	155 ± 15	$Cm/Ca(OH)_2$
	619.3	619.4, 619.9, 620.4 (t)	198 ± 16.0	C-S-H main band
			789 ± 42	
	621.5	621.5, 622.1, 622.7 (t)		C-S-H main band

(d) = double splitting, (t) = triple splitting

Table S2
$CaCO_3$ phases present after leaching of a Cm(III)-free C-S-H gel with a C/S ratio of 1.0 in dependence on the leaching solution and leaching time. Percentage amounts of formed phases are given in brackets.

Phase	0.02 M $NaHCO_3$					2.5 M NaCl/0.02 M $NaHCO_3$			
Day	1	4	8	30	Average	1	4	8	30
Calcite	✓ (72)	✓ (75)	✓ (52)	✓ (68)	67 ± 9	✓	✓	✓	✓
Vaterite	-	-	-	-		-	-	-	-
Aragonite	✓ (28)	✓ (25)	✓ (48)	✓ (32)	33 ± 9	-	-	-	-
Halite	-	-	-	-		✓	✓	✓	✓

Table S3
Theoretical calcium and silicon concentration in the leaching solution after a hypothetical complete dissolution of sample 1 and sample 2 at 10 g/L.

	Ca [mM]	Si [mM]
Sample 1 (C/S 1.0)	86.1	86.1
Sample 2 (C/S 2.0)	109.8	54.9

Table S4
Cm(III) emission band positions and lifetimes of sample 1 (C/S 1.0) at 10 K after leaching in 0.02 M $NaHCO_3$ or 2.5 M NaCl/0.02 M $NaHCO_3$.

Condition	λ_{ex} [nm]	Band positions [nm]	Lifetime ± 2σ [µs]	Species
$NaHCO_3$	605.2	621.3	1020 ± 27	C-S-H hot band
14 d	609.2	608.9, 609.2, 609.6 (t), 621.6 (l)	597 ± 44	Cm(III)/aragonite
	612.2	611.9, 612.5, 613.1 (t), 620.7 (l)	734 ± 55	Cm(III)/aragonite
	621.4	621.4, 621.9, 622.7 (t)	904 ± 19	C-S-H main band
NaCl/$NaHCO_3$	605.2	621.2	991 ± 38	C-S-H hot band
14 d	608.0	608.0 (l), 612.8, 613.5 (d), 615.2 (l), 622.0 (l)		Cm(III)/aragonite
	612.8	612.7, 613.3, 614.0 (t), 615.2 (l)	652 ± 23	Cm(III)/aragonite
	621.4	621.4, 621.9, 622.7 (t)	843 ± 24	C-S-H main band
NaCl/$NaHCO_3$	605.2	621.5	990 ± 32	C-S-H hot band
60 d	608.0	608.1 (l) 621.4, 623.2 (d/l)		Cm(III)/aragonite
	612.8	612.7, 613.4, 613.9 (t/l), 620.5 (l)		Cm(III)/aragonite
	621.4	621.4, 622.0, 622.7 (t)	854 ± 22	C-S-H main band

(d) = double splitting, (t) = triple splitting, (l) = low intensity

Table S5
$CaCO_3$ phases present after leaching of a Cm(III)-free C-S-H gel with a C/S ratio of 2.0 in dependence on the leaching solution and leaching time. Percentage amounts of formed phases are given in brackets.

Phase	0.02 M $NaHCO_3$					2.5 M NaCl/0.02 M $NaHCO_3$			
Day	1	4	8	30	Average	1	4	8	30
Calcite	✓ (87)	✓ (87)	✓ (82)	✓ (80)	84 ± 3	✓	✓	✓	✓
Vaterite	✓ (13)	✓ (13)	✓ (18)	✓ (20)	16 ± 3	-	-	-	-
Aragonite	-	-	-	-		-	-	-	-
Halite	-	-	-	-		✓	✓	✓	✓

Table S6
Cm(III) emission band positions and lifetimes of sample 2 (C/S 2.0) at 10 K after leaching in 0.02 M $NaHCO_3$ or 2.5 M NaCl/0.02 M $NaHCO_3$.

Condition	λ_{ex} [nm]	Band positions [nm]	Lifetime ± 2σ [μs]	Species
$NaHCO_3$	605.4	620.2, 621.2 (d)	908 ± 40	C-S-H hot band
14 d	609.2	621.7 (l)		C-S-H main band
	612.0	612.1 (l)		Cm(III)/$Ca(OH)_2$
		620.3, 621.7 (d/l)		C-S-H main band
	620.2	620.2, 620.7, 621.4 (t)	875 ± 24	C-S-H main band
	621.4	621.4, 621.9, 622.6 (t)	875 ± 26	C-S-H main band
NaCl/$NaHCO_3$	605.4	617.8, 620.7 (d)	857 ± 21	C-S-H hot band
14 d	612.8	612.8	312 ± 39	Cm(III)/$Ca(OH)_2$
		619.5, 620.5 (d)		C-S-H main band
	620.0	620.0, 620.5, 621.2 (t)	137 ± 56	C-S-H main band
			860 ± 27	
	621.4	621.4, 621.9, 622.7 (t)	161 ± 92	C-S-H main band
			904 ± 44	
NaCl/$NaHCO_3$	605.4	620.6	948 ± 44	C-S-H hot band
60 d	612.8	612.8 (l)		Cm(III)/$Ca(OH)_2$
		620.6 (l)		C-S-H main band
	620.0	620.0, 620.5, 621.2 (t)	802 ± 16	C-S-H main band
	621.4	621.4, 621.9, 622.7 (t)	852 ± 20	C-S-H main band

(d) = double splitting, (t) = triple splitting, (l) = low intensity

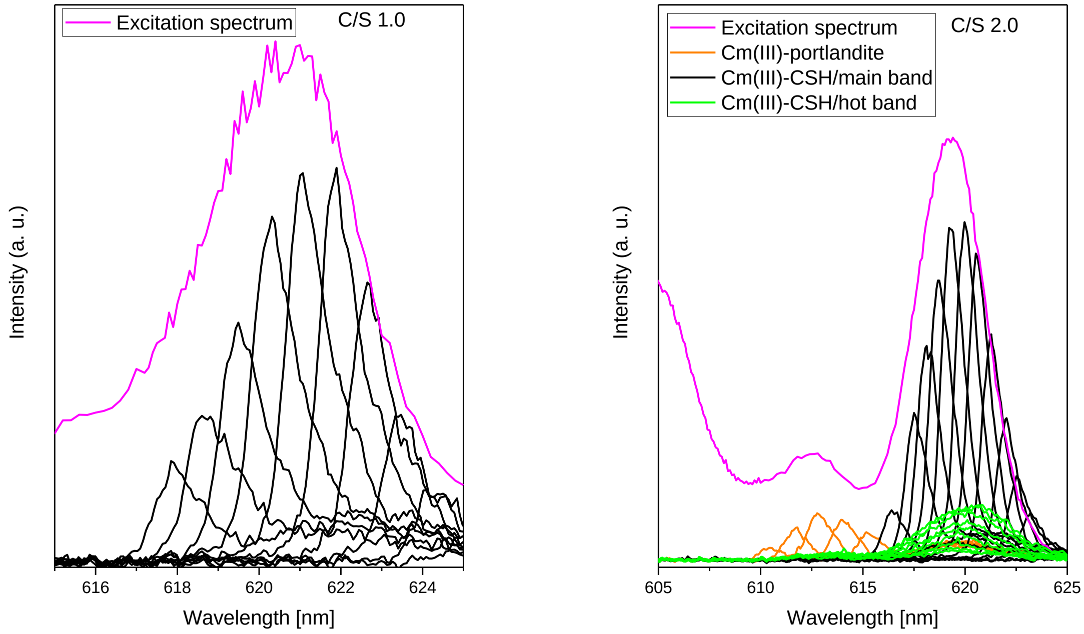

Fig. S1. Excitation spectra with associated emission bands (100 accumulations) of sample 1 (C/S 1.0) (left) and sample 2 (C/S 2.0) (right). Emission bands due to direct excitation of the Cm(III)/C-S-H main band between 618 and 624 nm (left, black). Emission bands due to direct excitation of the Cm(III)/C-S-H hot band (right, 603-608 nm, green), Cm(III)/C-S-H main band (right, 618-624 nm, black) and Cm(III) sorbed on portlandite (right, 610-615 nm, orange).

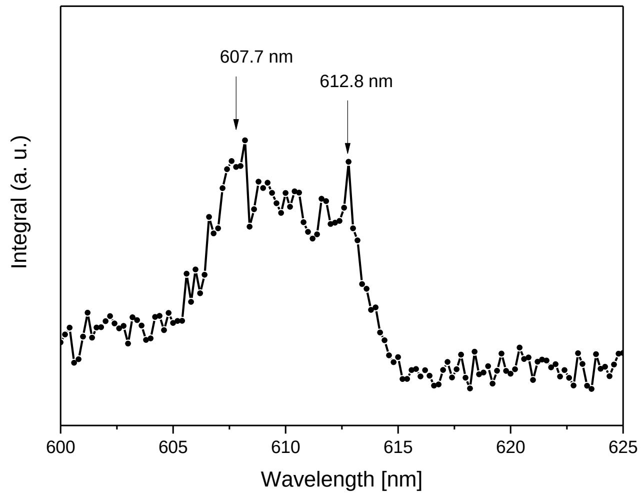

Fig. S2. Detailed view of the excitation spectrum C in Fig. 5 (left).
